# Mucins and Truncated *O*-Glycans Unveil Phenotypic Discrepancies between Serous Ovarian Cancer Cell Lines and Primary Tumours

**DOI:** 10.3390/ijms19072045

**Published:** 2018-07-13

**Authors:** Ricardo Coelho, Lara Marcos-Silva, Nuno Mendes, Daniela Pereira, Catarina Brito, Francis Jacob, Catharina Steentoft, Ulla Mandel, Henrik Clausen, Leonor David, Sara Ricardo

**Affiliations:** 1 Instituto de Investigação e Inovação em Saúde (i3S), Universidade do Porto, 4099-002 Porto, Portugal; rjcoelho@ipatimup.pt (R.C.); laras@ipatimup.pt (L.M.-S.); nmendes@ipatimup.pt (N.M.); danismpereira@gmail.com (D.P.); ldavid@ipatimup.pt (L.D.); 2 Institute of Molecular Pathology and Immunology of the University of Porto (IPATIMUP), 4099-002 Porto, Portugal; 3 Faculty of Medicine, University of Porto, 4099-002 Porto, Portugal; 4 Instituto de Biologia Experimental e Tecnológica (iBET), 2780-901 Oeiras, Portugal; anabrito@ibet.pt; 5 Instituto de Tecnologia Química e Biológica (ITQB) António Xavier, Universidade Nova de Lisboa, 2780-157 Oeiras, Portugal; 6 Glyco-Oncology, Ovarian Cancer Research, Department of Biomedicine, University Hospital Basel and University of Basel, 4031 Basel, Switzerland; francis.jacob@unibas.ch; 7 Copenhagen Center for Glycomics, Department of Odontology, Faculty of Health Sciences, University of Copenhagen, DK-2200 Copenhagen, Denmark; steentoft@sund.ku.dk (C.S.); ulma@sund.ku.dk (U.M.); hclau@sund.ku.dk (H.C.)

**Keywords:** serous ovarian carcinomas, ovarian cancer cell lines, MUC16, MUC1, truncated *O*-glycans, COSMC

## Abstract

Optimal research results rely on the selection of cellular models capable of recapitulating the characteristics of primary tumours from which they originate. The expression of mucins (MUC16 and MUC1) and truncated *O*-glycans (Tn, STn and T) represents a characteristic footprint of serous ovarian carcinomas (SOCs). Therefore, selecting ovarian cancer (OVCA) cell lines that reflect this phenotype is crucial to explore the putative biological role of these biomarkers in the SOC setting. Here, we investigated a panel of OVCA cell lines commonly used as SOC models, and tested whether, when cultured in 2D and 3D conditions, these recapitulate the mucin and *O*-glycan expression profiles of SOCs. We further explored the role of truncating the *O*-glycosylation capacity in OVCAR3 cells through knockout of the COSMC chaperone, using in vitro and in vivo assays. We found that the majority of OVCA cell lines of serous origin do not share the mucin and truncated *O*-glycan footprint of SOCs, although 3D cultures showed a higher resemblance. We also found that genetic truncation of the *O*-glycosylation capacity of OVCAR3 cells did not enhance oncogenic features either in vitro or in vivo. This study underscores the importance of well-characterized cellular models to study specific features of ovarian cancer.

## 1. Introduction

Cancer cell lines have been used for decades as elective in vitro models in cancer research. Despite the ubiquitous use of cancer cell line models, one question remains since the first cell line was isolated in the 1950s [1]: How well do in vitro cell line models reproduce the tumour features? The selection of well-characterized cancer cell lines that better mimic the tumour of origin is crucial to deliver precise results. Several studies have pointed out the need for good cell line models of the distinct histological subtypes of ovarian cancer (OVCA) [2,3]. Epithelial ovarian cancer is a highly heterogeneous disease [4], with serous ovarian carcinoma (SOC) being responsible for ~70% of epithelial ovarian cancers [5]. Unfortunately, we have imprecise/conflicting information on the histological subtype of the original primary tumour for the most commonly used OVCA cell lines, which limits proper selection of cell lines for studies [6]. A comparative study based on copy-number, mutations, and mRNA expression profiles revealed that most OVCA cell lines widely used as SOC models do not reproduce the molecular features of serous origin [7]. Another important aspect of in vitro cell models is the culture conditions, where 3D cell culture systems are now widely accepted as more representative models that better reflect gene and protein expression patterns as well as microRNA and metabolic profiles of the tumours, as compared to 2D monolayer cultures [8,9].

Previously, we showed that most SOC express mucins MUC16 and MUC1 and truncated *O*-glycans, Tn (GalNAcα1-*O*-Ser/Thr), STn (NeuAcα2–6GalNAcα1-*O*-Ser/Thr) and T (Galβ1–3GalNAcα1-*O*-Ser/Thr [10]. Furthermore, we identified the expression of Tn and STn as being highly specific for these malignant conditions. Here, we investigated whether the OVCA cell lines commonly used as SOC models, cultured in 2D and 3D conditions, reproduce the mucin and *O*-glycan expression profiles of SOCs. Additionally, we explored whether the expression of Tn and STn glycoforms has an impact on the biological behaviour of OVCA cells. To this end we used a previously established isogenic cell model of OVCAR3 SimpleCells (SC) [11]. OVCAR3 SC were genetically engineered to express truncated Tn and STn *O*-glycans. This was archived by knocking out the *COSMC* gene [12], a private chaperone for the core1 *O*-glycan elongation enzyme C1GalT1 [13,14], thus generating homogenous expression of Tn and STn truncated *O*-glycans. The models generated were tested using in vitro and in vivo assays. 

We found that the majority of OVCA cell lines of serous origin do not express the characteristic mucin and *O*-glycan footprint of SOCs. Moreover, we found that genetic truncation of *O*-glycosylation in OVCAR3 cells expressing MUC16 and MUC1 did not enhance oncogenic features in vitro or in vivo in xenografts. Our study underscores the importance of well-characterized cellular models to study specific features of ovarian tumours.

## 2. Results

### 2.1. Expression of MUC16, MUC1, and Truncated O-Glycans in a Panel of Ovarian Cancer Cell Lines

**Figure 1:**
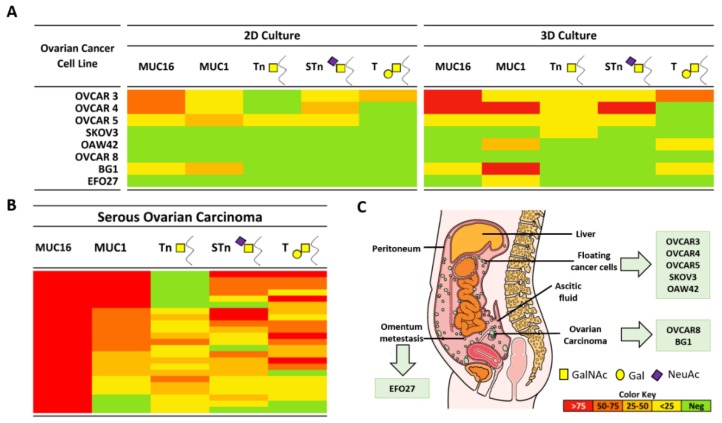
Expression of MUC16, MUC1, and truncated *O*-glycans in a panel of ovarian cancer cell lines. (**A**) Expression profile of MUC16, MUC1, Tn, STn, and T by immunocytochemistry in eight OVCA cell lines cultured in 2D and 3D conditions; (**B**) Expression pattern of the same mucins and truncated *O*-glycans in a series of 23 SOCs previously reported by us [10]; the colour key represents the percentage of positive cells for each marker; (**C**) Representation of the origin of ovarian cancer cell lines (adapted from © Macmillan Cancer Support 2018).

We studied the expression of mucins MUC16 and MUC1 and truncated *O*-glycans Tn, STn and T by immunocytochemistry in eight OVCA cell lines derived from different peritoneal cavity sites, in 2D and 3D culture conditions (Figure 1A). The expression profile of studied mucins and truncated *O*-glycans was not associated with the site of origin of OVCA cell lines (Figure 1C and Table S1). For example, OVCAR8 (derived from primary tumour in the ovary), SKOV3 (derived from ascitic fluid) and EFO27 (derived from omentum metastasis) presented the same immunophenotype without expression of MUC16, MUC1, Tn, STn, and T.

The number of positive cell lines and the percentage of positive cells for the studied biomarkers (MUC16, MUC1, Tn, STn, and T) was in general higher in 3D cultures. MUC16 was expressed in a higher number of cells from cell lines OVCAR3 and OVCAR4 in 3D cultures, but no differences were observed regarding the number of positive cell lines. MUC1 expression was present in 4/8 (50.0%) cell lines in 2D cultures and in 6/8 (75.0%) cell lines in 3D cultures. Truncated *O*-glycan expression in 2D cultures was 1/8 (12.5%) for Tn, 3/8 (37.5%) for STn, and 1/8 (12.5%) for T. In 3D cultures, the expression of Tn and T increased 3- to 4-fold compared with 2D cultures. For STn, we only observed an increase in the numbers of cell stained for STn in an OVCAR4 cell line.

**Figure 2:**
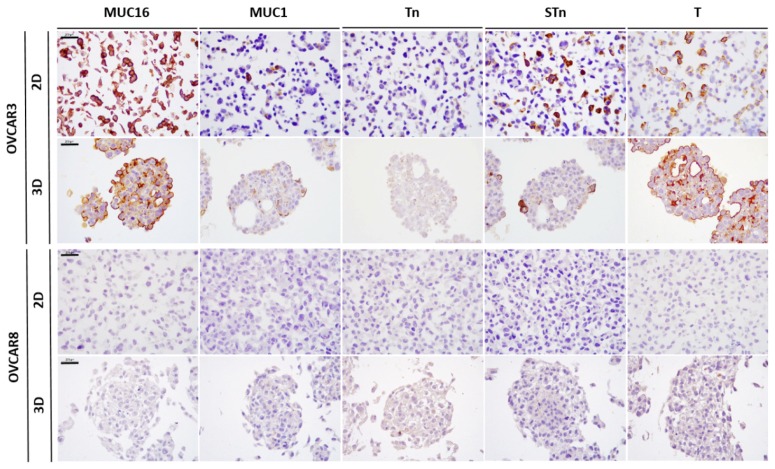
Expression of MUC16, MUC1, Tn, STn, and T in OVCAR3 and OVCAR8 cell lines, cultured in 2D and 3D conditions, assessed by immunocytochemistry. Scale bar, 200 µm.

Expression levels in 3D cultures were lower than those presented by tumour tissues. In the 23 SOCs previously studied by us [10] (Figure 1B), MUC16 expression was observed in 23/23 (100%) tumours, MUC1 in 20/23 (87.0%), and truncated *O*-glycans expression was present in 18/23 (78.3%) for Tn, 21/23 (91.3%) for STn, and 20/23 (87.0%) for T [10]. In the same paper, a validation series of 55 cases from a different hospital was analysed, and similar results were obtained for the footprint profile of SOC [10]. From the cell lines studied, the OVCAR3, OVCAR4, and OVCAR5 cell lines were the ones presenting a pattern of expression closer to the SOCs. On the other hand, OVCAR8 was negative for all evaluated mucins and *O*-glycans (Figure 2). Additionally, using a different 3D culture method with continuous agitation [15], we observed an increased percentage of positive cells for tested markers within each cell line, but no differences were observed regarding the number of positive and negative cell lines (Figure S1).

### 2.2. Truncated O-Glycans Reduce Proliferation while Increasing Apoptosis and Cell Migration

In order to explore the role of Tn and STn expression in OVCA, we used an isogenic OVCAR3 SC cell line model [11], which is genetically engineered to have impaired *O*-glycan elongation by KO of the core1 synthase chaperone, COSMC, resulting in homogeneously truncated Tn and STn *O*-glycans expression (Figure S2). 

**Figure 3:**
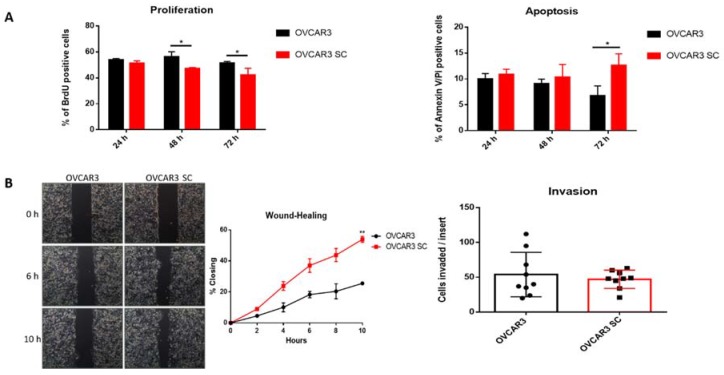
Truncated *O*-glycans affect the biological behaviour of OVCAR3 cells. (**A**) Flow cytometry analysis of cell proliferation (left) and apoptosis (right), quantified by BrdU and Annexin V/Pi positive cells, respectively. Significantly decreased proliferation was observed in OVCAR3 SC compared with parental OVCAR3 at 48 and 72 h after seeding. Significantly increased apoptosis was observed in OVCAR3 SC compared with parental OVCAR3 at 72 h after seeding; (**B**) Migratory (left) and invasive (right) properties of OVCAR3 and OVCAR3 SC, quantified following wound-healing assays and invasion through Matrigel, respectively. Significantly increased migration was observed in OVCAR3 SC compared with parental OVCAR3. No significant differences were observed in the invasive properties of OVCAR3 SC compared with parental OVCAR3. All experiments were performed in triplicate in at least two independent experiments. Values represent the mean ± SD, * *p* < 0.05, ** *p* < 0.01.

Effects of COSMC KO on cell proliferation and apoptosis were analysed by flow cytometry analysis of BrdU and Annexin V/Pi positive cells, respectively. As shown in Figure 3A, a significant decrease in cell proliferation and increase in apoptosis were observed in OVCAR3 SC compared with parental OVCAR3. To determine whether the homogeneous expression of Tn and STn modified the capacity of OVCAR3 cells to migrate, we performed wound healing assays (random cell motility). A significant increase in random cell migration was observed in OVCAR3 SC compared with parental OVCAR3 cells (Figure 3B). We also evaluated the invasive properties (directed cell motility) in both cell lines using the transwell chamber assay containing an extracellular matrix layer (Matrigel). As shown in Figure 3B, no significant differences were observed between OVCAR3 SC and the corresponding parental cell line.

### 2.3. O-Glycosylation Capacity Affects the Mesothelial Clearance of OVCAR3

**Figure 4:**
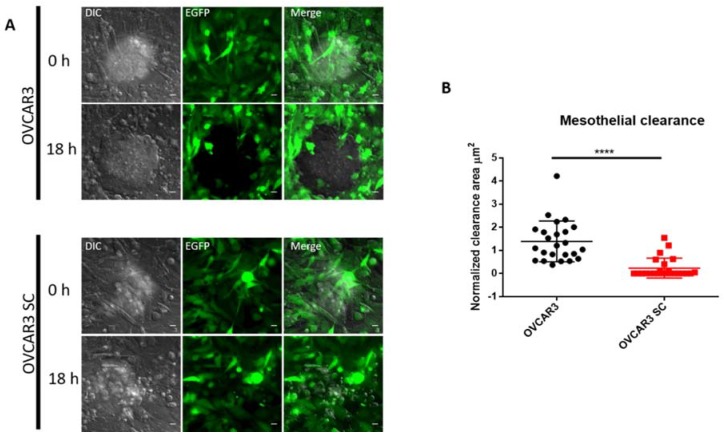
*O*-glycosylation capacity affects mesothelial clearance of OVCAR3. (**A**) Representative images from mesothelial clearance assays of OVCAR3 (upper) and OVCAR3 SC (bottom), taken at 0 and 18 h of co-culture; (**B**) Quantification of mesothelial clearance in parental OVCAR3 and OVCAR3 SC with a significant decrease of mesothelial clearance in OVCAR3 SC. The clearance area was measured by co-culturing aggregates of ovarian cancer cells with MeT5A-EGFP mesothelial cell monolayers. After 18 h of co-culture, the negative space created in the mesothelial monolayer by the ovarian cancer aggregates was measured and divided by the initial size of the ovarian cancer aggregates at time 0 to determine the normalized clearance area. More than eight aggregates were scored over three independent experiments. Scale bar, 20 µm. Values represent the mean ± SD, **** *p* < 0.0001.

The formation of peritoneal implants depends on the ability of OVCA cell clusters to attach to organs within the peritoneal cavity, a process that requires adhesion to and invasion through the mesothelial cell layer covering these organs. Electron microscopy studies of OVCA nodules attached to peritoneal cavity organs revealed that mesothelial cells are not present directly under the tumour mass, suggesting mesothelial clearance from the area beneath the tumour [16,17]. Mesothelial clearance assays have been used as an in vitro co-culture model to evaluate the ability of OVCA aggregates to attach and spread on mesothelial monolayers [18,19]. To investigate if the homogenous expression of Tn and STn *O*-glycans alters the mesothelial clearance capacity of OVCA aggregates, we co-cultured aggregates of parental OVCAR3 and SC with monolayers of mesothelial cells stably expressing EGFP protein (MeT5A-EGFP). As shown in Figure 4 and Supplementary Videos S1 and S2, OVCAR3 SC cleared mesothelial cells significantly less efficiently compared to the clearance achieved by parental OVCAR3 cells. We also observed that OVCAR3 SC have less ability to form well-defined aggregates compared with parental OVCAR3 (Figure S3), which can be related to the decreased mesothelial clearance observed in OVCAR3 SC. These data suggest that elongated *O*-glycans play a role in aggregate formation and the ability of OVCAR3 cells to breach the mesothelium.

### 2.4. OVCAR3 Xenografts Formed Larger Tumours than OVCAR3 SC

To analyse the effect of altered *O*-glycosylation in OVCAR3 on tumour formation, tumour growth, and invasive capacity, we established intraperitoneal (i.p.) xenografts of parental OVCAR3 and OVCAR3 SC. After eight weeks, some mice presented abdominal distension, which predicts peritoneal carcinomatosis and/or ascites, and all mice were sacrificed. After necropsies, abdominal tissues were harvested and fixed in formalin, and histological processing was performed for microscopic analysis of tumour growth and invasiveness. Haematoxylin and eosin (H&E) of the uterus and ovaries, peritoneal wall, pancreas, liver, peripheral lymph nodes, and lungs were examined as typical locations for tumour implants and metastasis.

**Figure 5:**
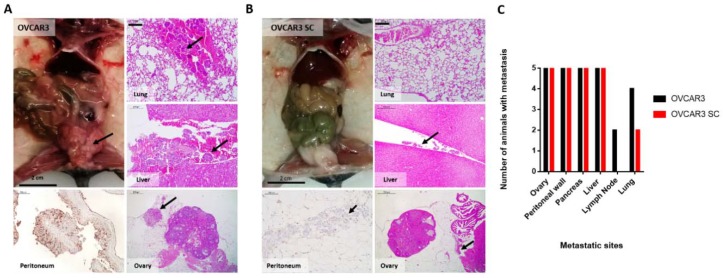
OVCAR3 xenografts formed larger tumours than OVCAR3 SC. (**A**) OVCAR3 xenografts formed large tumours in the peritoneum and in the Douglas Sac (arrow). Representative metastatic sites shown by H&E stain of lung, liver, and ovary. Immunocytochemistry showing the expression of T antigen of parental OVCAR3 cells in the peritoneum. Arrows indicate areas of tumour aggregates; (**B**) OVCAR3 SC xenografts formed smaller tumours compared with parental OVCAR3. Representative metastatic sites shown by H&E stain of lung, liver, and ovary. Immunocytochemistry showing the absence of T antigen expression of OVCAR3 SC cells in the peritoneum. Arrows indicate areas of tumour aggregates; (**C**) The number of animals with metastasis in the ovary, peritoneum, pancreas, and liver capsule was the same for both cell lines (5/5). OVCAR3 were identified in the lymph nodes of 2/5 mice and in the lungs of 4/5 mice. OVCAR3 SC were absent in the lymph nodes and present in the lung of 2/5 mice. A total of five mice were used per condition. The scale bar on microscopic images represents 200 µm.

Both parental OVCAR3 and SC were efficient to form tumours in mice. Regarding the size of the tumours, parental OVCAR3 xenografts presented macroscopically perceptible tumours in the peritoneal cavity (Figure 5A), while OVCAR3 SC only presented microscopic tumours (Figure 5B). The majority of tumour implants was found to be adherent to the surface of peritoneal organs (the ovaries, peritoneal wall, pancreas, and liver) in xenografts of both cell lines. Metastases within the peritoneal cavity were observed in the same number of animals and localized in the ovary, peritoneum, pancreas, and liver capsule for both parental OVCAR3 and SC (5/5). However, lymphatic/haematogenous metastases of parental OVCAR3 cells were identified in the lymph nodes of 2/5 xenografts and in the lung of 4/5 xenografts, whereas OVCAR3 SC cells were only identified in the lungs of 2/5 mice (Figure 5C).

## 3. Discussion

Expression of the mucins MUC16 and MUC1 in combination with the truncated *O*-glycans Tn, STn, and T is a phenotype observed in ~70% of SOCs [10]. In order to analyse and compare the expression of these biomarkers in tumour tissues retrieved from pathology archives with the expression in cultured cell lines, it is essential to clarify possible discrepancies arising from different pre-analytical conditions (fixation and histological processing methods) that may influence protein and *O*-glycan detection [20,21]. Therefore, we used formalin-fixed and paraffin-embedded cell lines for the immunocytochemistry procedure. Our results showed that within the panel of OVCA cell lines studied, OVCAR3, OVCAR4, and OVCAR5 best represented the characteristic footprint of mucins and truncated *O*-glycans found in SOCs. Strikingly, only OVCAR3 and OVCAR4 expressed MUC16 at levels similar to SOCs (i.e., >75% of cancer cells stained). In the literature there are few studies reporting on MUC16 expression in OVCA cell lines. In two of these studies, results were obtained by flow cytometry using OVCAR3 and SKOV3 cell lines and corroborate our results regarding MUC16 expression [22,23]. A larger OVCA cell line study was performed by Lee et al. [24], wherein the expression of commonly used biomarkers to identify different carcinoma histological subtypes in 31 cell lines was assessed in 2D and 3D conditions. The MUC16 expression results obtained by immunocytochemistry are concordant with our findings, reporting a percentage of less than 30% of epithelial ovarian cancer cell lines positive for MUC16 [24]. MUC16 expression is a hallmark of ovarian neoplasms of serous origin, widely used in serum detection assays as a marker of disease progression and response to therapy in the OVCA setting [25]. Importantly, truncated *O*-glycans are selectively found on circulating MUC16 in cancer patients and not in benign conditions [26]. The reason for a loss of MUC16 expression in most OVCA cell lines is unknown, but this is an observation that needs to be taken into account when using cell lines as surrogates for primary SOCs.

The pattern of expression of mucins and truncated *O*-glycans observed in this panel of OVCA cell lines was not related to the reported site of origin of each cell line since similar features were observed in OVCA cell lines generated from cells collected in different peritoneal cavity sites. Since “solid tumours” and floating cell clusters present in the ascitic fluid have different protein expression profiles and are at different stages of the metastatic process [27], it would be anticipated that cell lines derived from solid tumours (primary tumours or peritoneal implants) or from the peritoneal fluid would mirror the characteristics from the site of origin. Using an innovative microfluidic chip, Peterson et al. [28] found that several markers present in solid tumours had different expression levels in cancer cells present in the peritoneal fluid. For example, they found low levels of certain markers that have been gaining traction as drug targets (EphA2), or that have been touted as specific for (MUC16 and FOLR1) or overabundant (Mesothelin) in ovarian cancer [28]. 

Another parameter that we evaluated was the influence of different culture conditions in the expression levels of mucins and truncated *O*-glycans. Three-dimensional cultures are now widely accepted as cellular models of intermediate complexity between in vivo and in vitro monolayers [24,29]. In our study, MUC1 and T expression were higher in 3D than in 2D cultures, with two OVCA cell lines shifting from negative in 2D to positive in 3D conditions for both markers. In fact, by reproducing some of the morphological and functional features of the original tissue, 3D cultures have been used as a better cellular model to predict epithelial ovarian cancer outcomes [24,30]. Still, our results showed that the expression pattern of OVCA cell lines cultured in 3D is far from that presented by SOC regarding MUC16, MUC1, Tn, STn, and T expression. We are now at work on prospective studies to evaluate paired samples of OVCA and cancer cell aggregates in the peritoneal fluid, where we will also assess the effect of culturing cells for short and long time periods.

Additionally, it will be relevant to perform peritoneal implantation of primary tumour tissue samples for comparison with our current cell line models. 

The expression of Tn and STn truncated *O*-glycans is widely reported as a cancer-associated phenotype in several human cancers, including OVCA, with prognostic implications [31,32,33,34,35]. However, the effects of expression of truncated Tn and STn *O*-glycans in OVCA is poorly explored. Since most cell lines that we evaluated lack the truncated *O*-glycan signature of SOC, we decided to use a genetically engineered cell model to study effects. We demonstrated that OVCAR3 SC exhibit decreased cell proliferation and increased apoptosis compared to parental OVCAR3 cells. These findings are in agreement with the results by Chou et al. [36], who found that the knockdown of the C1GalT1 enzyme leads to decreased growth of OVCA cells [36]. Moreover, we found that OVCAR3 SC, despite increased cell migration, also exhibit a reduction in the mesothelial clearance capacity and form smaller and less invasive tumours in the mice model. Several studies reported that the expression of STn truncated *O*-glycans is more frequently observed at the invasive front of ovarian tumours, but less often in metastatic lesions [34,37]. Thus, we speculate that the expression of Tn and STn *O*-glycans facilities the detachment of individual cells from the primary ovarian tumour by reducing cell–cell aggregation (Figure S3); however, this may not improve the settlement of metastatic cells at secondary sites. On the contrary, Radhakrishnan and co-workers, using a similar strategy in a pancreatic cancer cell line (T3M4), found that the T3M4 SC with truncated O-glycans exhibited enhanced proliferation and oncogenic features, including enhanced invasive properties in culture, and enhanced growth and invasion in xenografts [38]. The differences in effects in the two model systems may reflect the differences in tumour type or cell lines. In the particular case of ovarian cancer, where peritoneal dissemination is seeded mainly by transcelomic homing of tumour aggregates, the role of truncated *O*-glycans can be diverse. 

Optimal research results rely on the selection of cellular models capable of reproducing the characteristics of the tumour of origin. The expression of MUC16, MUC1, Tn, STn, and T is a footprint in SOC. Therefore, when studying mucins and truncated *O*-glycans, the selection of OVCA cell lines that reflect this phenotype is crucial to explore the putative role of each biomarker in the SOC setting. The very limited expression of MUC16 in the majority of SOC cell lines was remarkable since it represents the major ovarian cancer biomarker. Despite the inherent limitations of using cell lines as surrogate models of complex biological entities such as tumours, we showed that in 3D conditions cells presented an expression phenotype closer to tumours than cells in monolayers. Finally, we observed that the homogeneous expression of Tn and STn *O*-glycans in OVCA cells, using the *COSMC* KO strategy, did not intensify oncogenic features either in vitro or in vivo, in contrast to results observed in other tumour contexts.

## 4. Materials and Methods

### 4.1. Cell Lines Culture

Ovarian cancer cell lines were cultured under standard conditions in RPMI 1640 (Thermo Fisher Scientific, Waltham, MA, USA) containing 10% foetal bovine serum (FBS) (Biowest Nuaillé, France)). Normal immortalized mesothelial cell line MeT5A (ATCC, American Type Culture Collection) was maintained in Medium 199 (Thermo Fisher Scientific) containing 10% FBS, 3.3 nM epidermal growth factor (EGF) (PeproTech, London, UK), 400 nM hydrocortisone (Sigma, St. Louis, MO, USA), 870 nM Bovine insulin (Sigma) and 20 nM HEPES (Thermo Fisher Scientific). Cell lines were maintained at 37 °C and 5% CO_2_. All cell lines were authenticated using short tandem repeat (STR) profiling and regularly tested for the absence of mycoplasma. For the 3D cultures, polyHEMA (Poly(2-hydroxyethyl methacrylate)) (Sigma) coated plates were prepared by dissolving 120 mg/mL of polyHEMA in 95% ethanol, then adding 100 µL of the solution to 96-well round-bottom plates and drying for 48 h at 55 °C. Ovarian cancer aggregates were generated by plating 4 × 10^3^ cells per well and incubated for four days. 

### 4.2. Cell Microarray (CMA) Construction and Immunocytochemistry

2D cultures were collected by scraping cells from the flask with PBS 1× and 3D cultures were simply aspirated from each well, followed by centrifugation and fixation with 10% neutral-buffered formalin. After fixation, cell pellets were embedded in HistoGel (Thermo Fisher Scientific) according to the manufacturer’s instructions, followed by standard histological processing and paraffin embedding. Each cell line block (donor block) was sectioned and stained with haematoxylin and eosin (H&E) for morphology control. Cell microarray (CMA) was designed and constructed by adding one core (1.5 mm in diameter) from each donor block to a recipient paraffin block. Tumour tissue cores were included as controls. After construction, CMA was homogenized at 37 °C overnight and sectioned with a standard microtome at 3- to 4-µm thickness. After deparaffinization, heat-induced (98 °C) antigen retrieval was performed with a citrate buffer (pH 6.0) (Thermo Fisher Scientific), and slides were incubated with hydrogen peroxide 3%. CMAs were immunostained with monoclonal antibodies for MUC16 (5E11) [39] and M11 (Dako-Agilent, Santa Clara, CA, USA), MUC1 (HMFG2) [40], Tn (5F4) [41], STn (TKH2) [42], and T (3C9) [43]. Undiluted hybridoma culture supernatants (5E11, HMFG2, 5F4, TKH2 and 3C9) and M11 diluted at 1/60 in antibody diluent (Thermo Fisher Scientific) were incubated for 1 h at room temperature (RT). Primary antibodies were detected using a secondary antibody with HRP polymer (Dako) and visualization of the reaction was performed using diaminobenzidine according to the manufacturer’s instructions. Immunocytochemistry were evaluated by three independent observers (LD, SR, and RC), who registered cytolocalization of the staining and the percentage of cells stained (0–10%, >10–25%, >25–50%, >50–75%, and >75%). When less than 10% of cells were stained, cases were considered negative.

### 4.3. Generation of MeT5A Clones Stable Expressing EGFP Protein

The generation of MeT5A clones stably expressing EGFP protein was achieved by the transfection of the pEGFP-C1 vector (BD Biosciences, Franklin Lakes, NJ, USA) using Lipofectamine 2000 reagent (Thermo Fisher Scientific). Selection was initiated 48 h after transfection in a medium supplemented with 0.3 mg/mL of Geneticin (G418) (Thermo Fisher Scientific). Neomycin-resistant positive clones obtained through limiting dilution were routinely maintained with 0.1 mg/mL of G418. 

### 4.4. Apoptosis Assay

Cell apoptosis was measured by flow cytometry using the Annexin V-FITC apoptosis detection kit (Thermo Fisher Scientific) according to the manufacturer’s instructions. Briefly, cells were seeded in six-well plates at the density of 4 × 10^5^ cells per well and incubated for different times (24, 48, and 72 h). After the incubation time, floating and adherent cells were harvested, pelleted by centrifugation, and suspended in 400 μL of binding buffer. One aliquot of 195 μL of each sample was first stained with 5 μL of Annexin V-FITC, in the dark, for 10 min and then with 10 μL of propidium iodide (20 μg/mL). Samples were read in a BD Accuri™ C6 (BD Biosciences) flow cytometer, and analysis was performed using the FlowJo software (Ashland, OR, USA).

### 4.5. Proliferation Assay

Cell proliferation was evaluated by measuring 5-Bromo-2′-deoxyuridine (BrdU) incorporation during DNA synthesis, following the manufacturer’s instructions (BrdU labelling and detection kit 1, Roche, Basel, Switzerland). Briefly, cells were seeded in six-well plates at a density of 4 × 10^5^ cells per well and incubated for different times (24, 48, or 72 h). After the incubation time, cells were gently washed in PBS and BrdU was added to each well at a final concentration of 10 µM before incubating for 1 h at 37 °C. Immediately after incubation, the cells were harvested, washed with PBS, and fixed in ice-cold methanol for 30 min. This was followed by treatment with HCl (Thermo Fisher Scientific) at 4 M for 20 min. BrdU detection was performed using monoclonal antibody against BrdU (1/20) (Bu20a, Dako), diluted in PBS containing 0.5% Tween 20 (Sigma) and 0.05% BSA (Sigma) for 1 h at RT. After that, cells were further washed with PBS and incubated with the secondary antibody labelled with FITC (1/200, polyclonal rabbit anti-mouse immunglobulin/FITC, Dako) for 30 min at RT. Unstained cells and cells stained only with the secondary antibody were used as a control. Data acquisition was performed using a BD Accuri™ C6 (BD Biosciences) flow cytometer, and the analysis was performed using the FlowJo software.

### 4.6. Wound Healing Assays

The wound healing assays were performed using silicone culture inserts (Ibidi, Planegg, Germany). These silicone inserts contain two cell culture reservoirs, separated by a 500-µm wall. Briefly, the inserts were allowed to attach to the surface of the wells for 30 min at room temperature. Then, 6 × 10^4^ cells were plated in each reservoir and incubated for 24 h at 37 °C to reach confluence. After the incubation time, inserts were removed, followed by a gentle wash with PBS, and 2 mL of culture media supplemented with 5% FBS (Biowest) were added. Phase contrast images were acquired every 2 h over a 10-h period at 40× magnification using an Olympus CKX41 inverted microscope. The closing area was quantified using Fiji software (Madison, WI, USA).

### 4.7. Matrigel Invasion Assays

The Matrigel invasion assays were performed using BD BioCoat Matrigel invasion chamber inserts (BD Biosciences) according to the manufacturer’s instructions. Briefly, Matrigel-coated inserts were pre-incubated for 1 h with serum-free RPMI before adding the ovarian cancer cells. A total of 5 × 10^4^ cells were seeded in the upper chamber in 500 µL of RPMI supplemented with 1% FBS. In the lower compartment was added 750 µL of RPMI containing 10% FBS as a chemoattractant. Then, cells were incubated at 37 °C and allowed to invade through the Matrigel barrier for 24 h. Following incubation, non-invasive cells were removed with a cotton swab, whereas invasive cells were fixed in ice-cold methanol for 10 min. The membranes were carefully cut and mounted on microscope slides with Vectashield with DAPI (Vector, Burlingame, CA, USA). The number of invasive cells was counted in an inverted fluorescence microscope (Zeiss Axio Imager Z1, Oberkochen, Germany), and images were acquired using Axiovision software (Oberkochen, Germany) at 200× magnification. 

### 4.8. Mesothelial Clearance Assay

The mesothelial clearance assays were performed based on a protocol previously described [44] with minor alterations. MeT5A cells stably expressing EGFP protein were seeded in eight-well chambers (Ibidi) coated with 10 µg/mL of collagen type I (Millipore, Burlington, MA, USA) and 5 µg/mL of fibronectin (Sigma) and incubated for 24 h to form confluent monolayers. In parallel, aggregates of ovarian cancer cells were generated by seeding 350 cells per well in 96-well plates with a round bottom preciously coated with polyHEMA. After incubating for 24 h, the OVCA aggregates were transferred to the wells containing the mesothelial monolayers. Using the inverted motorized epifluorescence microscope Leica DMI 6000-time lapse, live cell imaging was performed for 24 h. To quantify the mesothelial clearance area, the non-fluorescent surface created by the invading ovarian cancer aggregate in the EGFP mesothelial monolayer was measured at 18 h of co-culture and divided by the initial two-dimensional area of the aggregate at the initial seeding time. All measurements were taken using the Fiji software. More than eight multicellular aggregates were imaged by each cell line in each experiment in a total of three biological experiments.

### 4.9. Establishment of I.P. Xenograft Models in Nude Mice

All procedures in animals were performed in accordance with the European Guidelines for the Care and Use of Laboratory Animals, Directive 2010/63/UE, Portuguese National Regulation published in 2013 (Decreto-Lei n.8 113/ 2013 de 7 de Agosto) and approved by the local Ethics Committee of the Instituto de Investigacão e Inovação em Saúde- i3S (Porto, Portugal). Project identification code 0421/000/000/2017, date (24/05/17). The authors involved in these experiments have an accreditation for animal research given from the Portuguese Veterinary Board (Ministerial Directive 1005/92). NIH(S)II: nu/nu mice, strain described by [45] were generated under Ipatimup supervision. Mice were monitored for two weeks and kept for an experimental period of eight weeks. To generate i.p. xenografts of OVCAR3 and OVCAR3 SC, 2 × 10^7^ cells in 200 µL of PBS were injected intraperitoneally in a total of five mice per cell line. Animals were monitored twice a week. At the end of the experimental period (eight weeks), mice were humanely euthanized (with anaesthesia followed by cervical dislocation) and necropsies performed. Animal organs were harvested for histological processing, embedding, and sectioning. H&E stain was performed in slides from all tissue blocks to evaluate tumour localization, growth, and invasion. 

### 4.10. Statistics

Each experiment was carried out in triplicate in at least two independent experiments, and data were expressed as mean ± SD. Statistical analysis was performed using Student’s *t*-test. A value of *p* < 0.05 was considered significantly different.

## Supplementary Materials

Supplementary materials can be found at http://www.mdpi.com/1422-0067/19/7/2045/s1.

## Abbreviations

SOC	Serous ovarian carcinoma
2D	Two-dimensional
3D	Three-dimensional
OVCA	Ovarian cancer
SC	Simple cells
PBS	Phosphate-buffered saline
SD	Standard deviation
EGFP	Enhanced green fluorescent protein
DAPI	4′,6-Diamidino-2-Phenylindole, Dihydrochloride
FBS	Fetal bovine serum
DNA	Deoxyribonucleic acid
FITC	Fluorescein isothiocyanate
BrdU	5-Bromo-2′-deoxyuridine
G418	Geneticin
CMA	Cell microarray
H&E	Haematoxylin and eosin
STR	Short tandem repeats
EGF	Epidermal growth factor
HRP	Horseradish peroxidase
C1GalT1	β1,3-galactosyltransferase
