# Supplementary material for: Mucins and Truncated O-Glycans Unveil Phenotypic Discrepancies between Serous Ovarian Cancer Cell Lines and Primary Tumours
Source: Int J Mol Sci. 2018 Jul 13;19(7):2045. doi: 10.3390/ijms19072045 (PMC6073732; doi:10.3390/ijms19072045)
Supplement: Supplementary file 1 [file ijms-19-02045-s001.zip › Supplementary Files/Table S1.pdf]

Supplementary: Mucins and Truncated *O*-Glycans Unveil Phenotypic Discrepancies between Serous Ovarian Cancer Cell Lines and Primary Tumours

**Ricardo Coelho [1,2,3], Lara Marcos-Silva [1,2,4,5], Nuno Mendes [1,2], Daniela Pereira [1,2,†], Catarina Brito [4,5], Francis Jacob [6], Catharina Steentoft [7], Ulla Mandel [7], Henrik Clausen [7], Leonor David [1,2,3]
and Sara Ricardo [1,2,3,*]**

1 Instituto de Investigação e Inovação em Saúde (i3S), Universidade do Porto, 4099-002 Porto, Portugal; rjcoelho@ipatimup.pt (R.C.); laras@ipatimup.pt (L.M.-S.); nmendes@ipatimup.pt (N.M.); danismpereira@gmail.com (D.P.) †; ldavid@ipatimup.pt (L.D.)
2 Institute of Molecular Pathology and Immunology of the University of Porto (IPATIMUP), 4099-002 Porto, Portugal
3 Faculty of Medicine, University of Porto, 4099-002 Porto, Portugal
4 Instituto de Biologia Experimental e Tecnológica (iBET), 2780-901 Oeiras, Portugal; anabrito@ibet.pt
5 Instituto de Tecnologia Química e Biológica (ITQB) António Xavier, Universidade Nova de Lisboa, 2780-157 Oeiras, Portugal
6 Glyco-Oncology, Ovarian Cancer Research, Department of Biomedicine, University Hospital Basel and University of Basel, 4031 Basel, Switzerland; francis.jacob@unibas.ch
7 Copenhagen Center for Glycomics, Department of Odontology, Faculty of Health Sciences, University of Copenhagen, Copenhagen, DK-2200, Denmark; steentoft@sund.ku.dk (C.S.); ulma@sund.ku.dk (U.M.); hclau@sund.ku.dk (H.C.)
* Correspondence: sricardo@ipatimup.pt; Tel.: +351-22-040-8800
† Present work address: Portsmouth Hospitals NHS Trust, United Kingdom

Table S1. Information on ovarian cancer cell lines retrieved from ExPASy, a Bioinformatics Resource portal (https://www.expasy.org).

Cell Line		Clinico-pathological Data				References	
**Name**	**Category**	**Origin**	**Age**	**Diagnosis**	**Treatment (before generation of cell line)**	1	2
**OVCAR3**	Cancer Cell line	Ovarian cancer (Ascites)	60Y	High grade ovarian serous adenocarcinoma	Chemotherapy (Cyclophosphamide, Adriamycin and cisplatin)	Cancer Res. 43(11):5379-89 (1983)	https://web.expasy.org/cellosaurus/CVCL_0465
**OVCAR4**	Cancer Cell line	Ovarian cancer (Ascites)	NA	High grade ovarian serous adenocarcinoma	Chemotherapy (Cyclophosphamide, Adriamycin and cisplatin)	Semin. Oncol. 11(3):285-298(1984)	https://web.expasy.org/cellosaurus/CVCL_1627
**OVCAR5**	Cancer Cell line	Ovarian cancer (Ascites)	NA	High grade ovarian serous adenocarcinoma	Untreated patient	J. Clin. Invest. 76:1261-1267(1985)	https://web.expasy.org/cellosaurus/CVCL_1628
**OVCAR8**	Cancer Cell line	Ovarian adenocarcinoma	NA	High grade ovarian serous adenocarcinoma	Chemotherapy (Carboplatin)	Int. J. Cancer 45:416-422(1990)	https://web.expasy.org/cellosaurus/CVCL_1629
**SKOV3**	Cancer Cell line	Ovarian cancer (Ascites)	63Y	Ovarian serous cystadenocarcinoma	ThioTEPA	J. Natl. Cancer Inst. 59:221-226(1977)	https://web.expasy.org/cellosaurus/CVCL_0532
**OAW42**	Cancer Cell line	Ovarian cancer (Ascites)	46Y	Ovarian cystadenocarcinoma	Chemotherapy (Cisplatin)	J. Natl Cancer Inst., 72, 513 (1984)	https://web.expasy.org/cellosaurus/CVCL_1615
**BG1**	Cancer Cell line	Ovarian Adenocarcinoma	NA	Ovarian adenocarcinoma	Not available	Cancer 63:280-288(1989)	https://web.expasy.org/cellosaurus/CVCL_6570
**EFO27**	Cancer Cell line	Ovarian cancer (Omentum metastasis)	36Y	Serous papillary denocarcinoma[1] Ovarian mucinous adenocarcinoma [2]	Not available	J. Natl. Cancer Inst. 70:839-845(1983)	https://web.expasy.org/cellosaurus/CVCL_1192
