# Supplementary figures and images for: Mucins and Truncated O-Glycans Unveil Phenotypic Discrepancies between Serous Ovarian Cancer Cell Lines and Primary Tumours
Source: Int J Mol Sci. 2018 Jul 13;19(7):2045. doi: 10.3390/ijms19072045 (PMC6073732; doi:10.3390/ijms19072045)

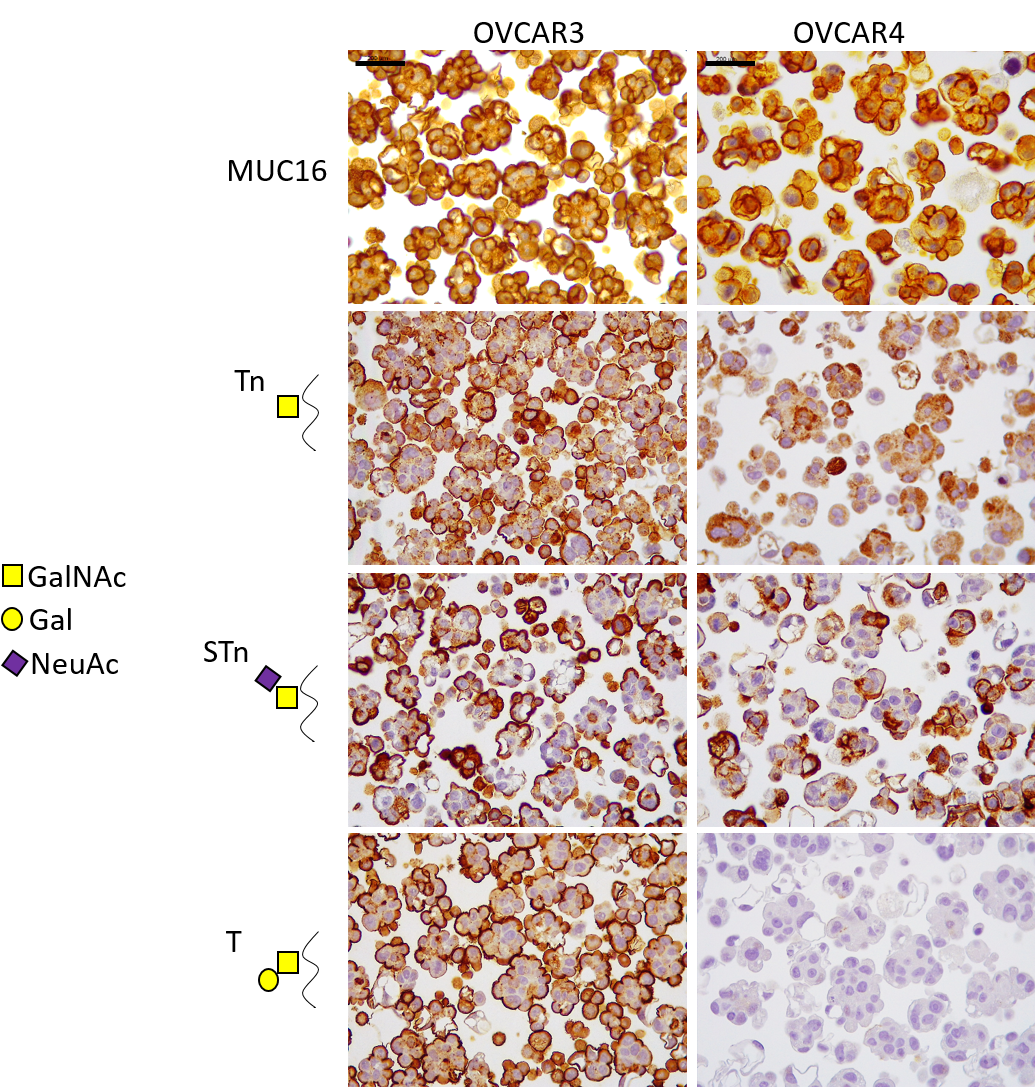

Supplement: Supplementary file 1 [file ijms-19-02045-s001.zip › Supplementary Files/Figure S1.tif]

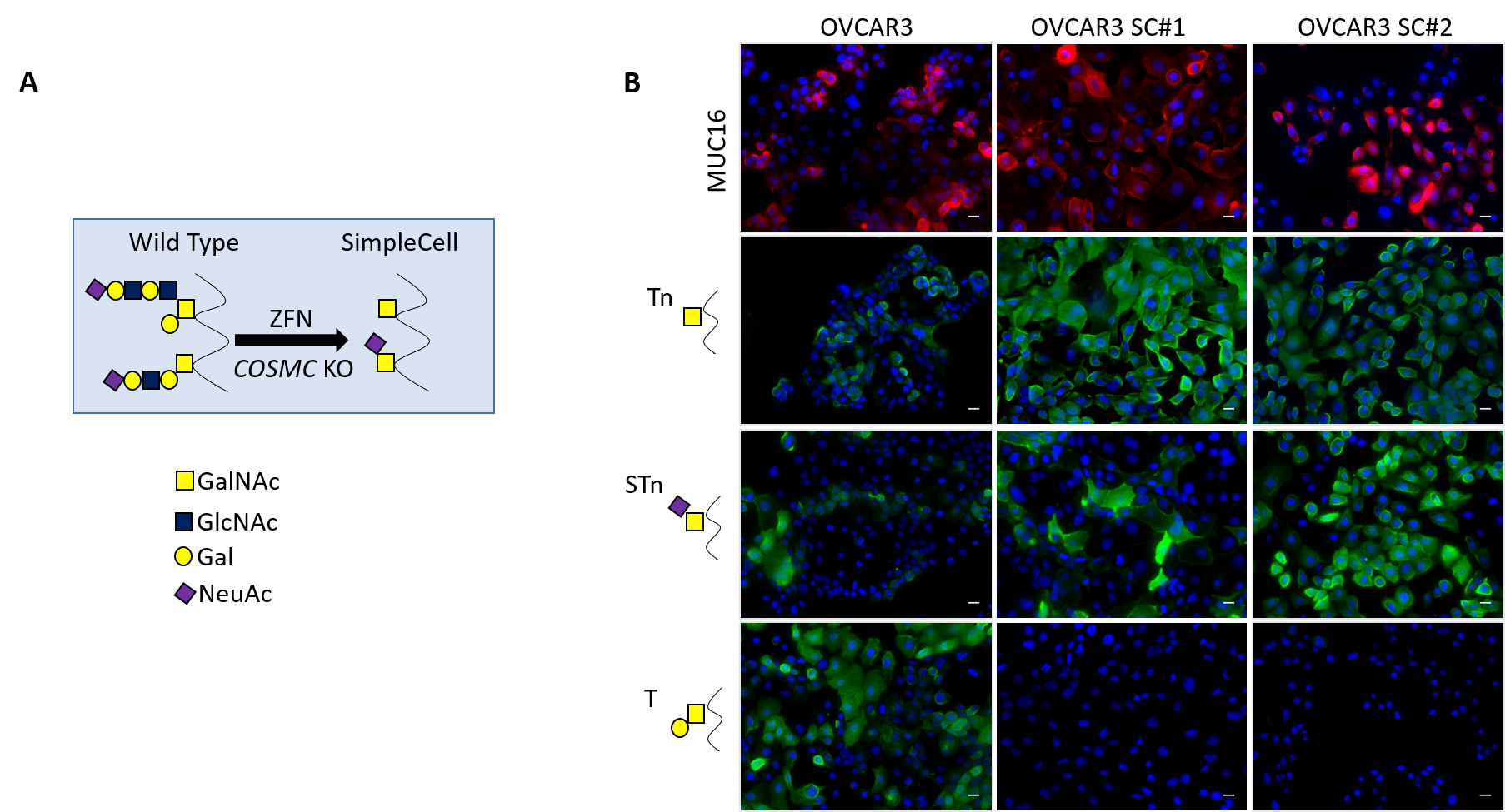

Supplement: Supplementary file 1 [file ijms-19-02045-s001.zip › Supplementary Files/Figure S2.tif]

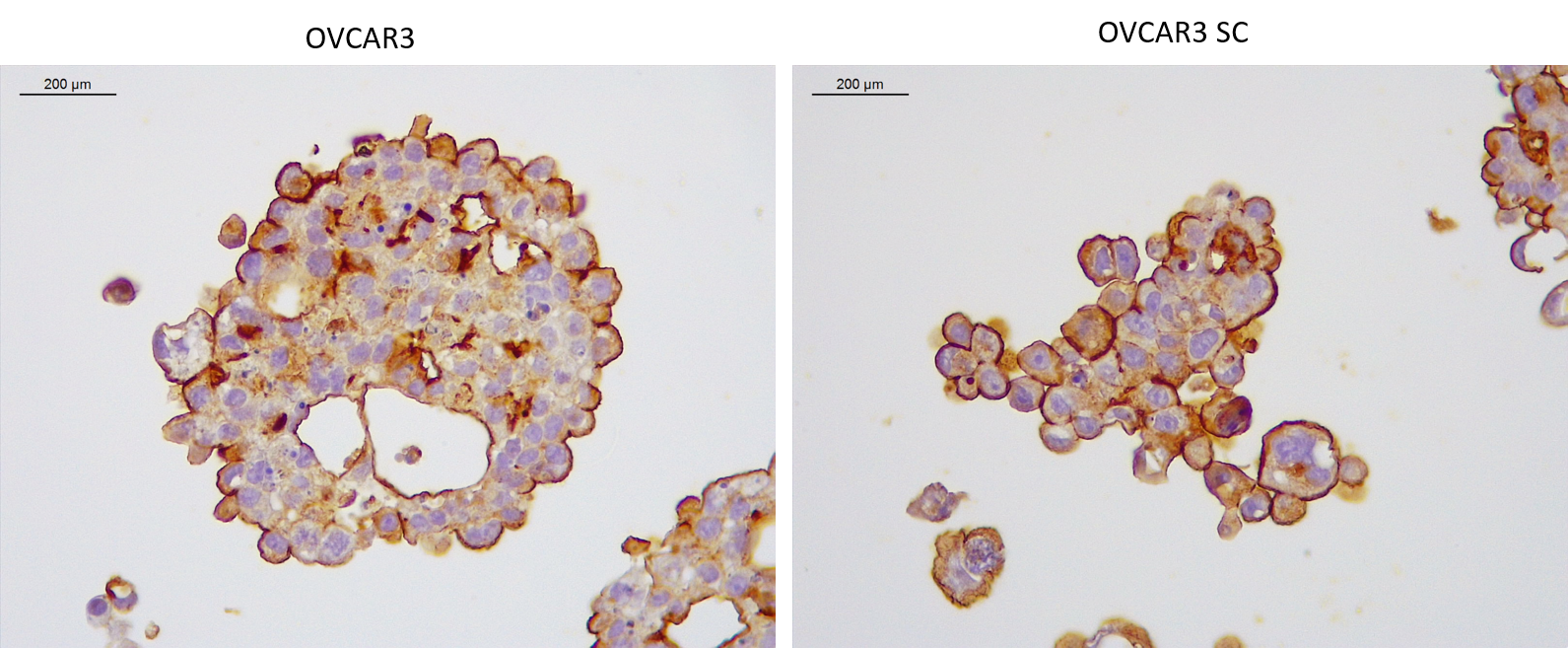

Supplement: Supplementary file 1 [file ijms-19-02045-s001.zip › Supplementary Files/Figure S3.tif]
